# Preclinical activity of MBM-5 in gastrointestinal cancer by inhibiting NEK2 kinase activity

**DOI:** 10.18632/oncotarget.12687

**Published:** 2016-10-15

**Authors:** Yanfen Fang, Yannan Kong, Jianbei Xi, Mengli Zhu, Tong Zhu, Tongtong Jiang, Brendan Frett, Wenhao Hu, Hong-yu Li, Mingliang Ma, Xiongwen Zhang

**Affiliations:** ^1^ Shanghai Engineering Research Center of Molecular Therapeutics and New Drug Development, College of Chemistry and Molecular Engineering, East China Normal University, Shanghai, China; ^2^ Department of Pharmaceutical Science, College of Pharmacy, University of Arkansas for Medical Sciences, Little Rock, AR, USA

**Keywords:** NEK2, mitosis, chromosome misalignment, cytokinesis failure, apoptosis

## Abstract

NEK2 is a conserved mitotic regulator critical for cell cycle progression. Aberrant expression of NEK2 has been found in a variety of human cancers, making it an attractive molecular target for the design of novel anticancer therapeutics. In the present study, we have identified a novel compound MBM-5, which was found to bind to NEK2 with high affinity by docking simulations study. MBM-5 potently inhibited NEK2 kinase activity *in vitro* in a concentration-dependent manner. MBM-5 also suppressed cellular NEK2 kinase activity, as evidenced by the decreased phosphorylation of its substrate Hec1 on S165 in a concentration- and time-dependent manner. This inhibition impeded mitotic progression by inducing chromosome segregation defects and cytokinesis failure; therefore leading to accumulation of cells with ≥4N DNA content, which finally underwent apoptosis. More importantly, MBM-5 treatment effectively suppressed the tumor growth of human gastric and colorectal cancer cells xenografts. Taken together, we demonstrated that MBM-5 effectively inhibited the kinase activity of NEK2 and showed a potential application in anti-cancer treatment regimens.

## INTRODUCTION

Never in Mitosis Related Kinase 2 (NEK2) is a member of the Never in Mitosis (NIMA) Related Kinases (NEKs) family that has a broadly structural similarity to the mitotic regulator NIMA of the filamentous fungus *Aspergillus nidulans* [1]. NEK2 is well recognized as a multifunctional serine/threonine kinase with key roles in cell cycle regulation, particularly with respect to the centrosome cycle. NEK2 localizes to the centrosome and triggers centrosome separation by phosphorylating centrosome cohesion proteins C-Nap1, Rootletin and Cep68 [2–4]. NEK2 also regulates microtubule organization and stabilization through phosphorylation of ninein-like protein (Nlp) [5]. Moreover, NEK2 operates a faithful kinetochore microtubule attachments by phosphorylation of highly expressed in cancer 1 (Hec1) [6, 7]. Through direct interaction with mitotic arrest deficient-like 1 (MAD1) or phosphorylation of Hec1 and Sgo1, NEK2 also modulates chromosome alignment and the spindle assembly checkpoint (SAC) signaling, thus regulating chromosome separation [8–10]. In addition, NEK2B (a splice variant of NEK2) is observed to be required for execution of mitotic exit as NEK2B depleted cells were unable to complete cytokinesis and resulted in the formation of multinucleated cells [11].

NEK2 has been reported to be overexpressed in a wide variety of human cancers, such as gastric cancer [12], colorectal cancer [13, 14], prostate cancer [15] and breast cancer [16, 17]. NEK2 overexpression is associated with tumorigenensis, tumor progression, drug resistance and predicts poor prognosis [18, 19]. Several preclinical studies using RNA interference targeting NEK2 have shown the efficient anti-tumor effect against different type of cancers. Suppression of the NEK2 expression with siRNA inhibited cell proliferation and induced cell death of breast cancer, cholangiocarcinoma, colorectal cancer, multiple myeloma, hepatoma and prostate cancer cells *in vitro*, and led to a reduction of tumor size in xenograft-nude mouse model [13, 15, 17, 20–23]. Moreover, silencing of NEK2 expression in breast cancer and colorectal cancer cells dramatically increased the susceptibility to chemotherapeutic drugs, such as paclitaxel, doxorubicin and cisplatin [13, 24]. These findings suggest NEK2 as a valuable anticancer target.

Development of NEK2 inhibitors has attracted much attention recently. The sunitinib-like oxindole inhibitor (SU11652), two thiophene-based PLK1 inhibitors and a series of viridian/wortmannin-like compounds were reported to have activity against NEK2, even though their activities against other kinases were greater [25–27]. Then a series of aminopyrazines and benzimidazoles were developed and showed activities against NEK2 kinase with IC_50_ of micromole range. However, none of them was active in cells because of insufficient membrane permeability [28, 29]. By combining key scaffold of the aminopyrazines and benzimidazoles, the hybrids finally yielded potent compounds with better activity against NEK2 (IC_50_=0.022-2.680 μM) and capable of modulating the phosphorylation of NEK2 substrates in cells [30]. Besides above reversible compounds, an irreversible inhibitor JH295 with IC_50_=0.770 μM against NEK2 was reported; however, its antitumor activity against cancer cell lines was not evaluated [31]. Moreover, the *in vivo* antitumor activity of any compound mentioned above has not been disclosed yet. Therefore, great efforts should be made to develop novel NEK2 inhibitors that are suitable for clinical applications.

By performing molecular docking analysis, we screened five hundred compounds from our in-house compound library to test their affinity to the ATP-site of the NEK2 kinase. Compounds with high docking score were selected to determine their potential activities against NEK2 kinase *in vitro*. A novel compound named MBM-5 was found to have the best inhibitory activity against NEK2 kinase. Further biologic testing confirmed that MBM-5 potently inhibited cellular NEK2 activity and exhibited antitumor activity *in vitro* and *in vivo*.

## RESULTS

### MBM-5 inhibits NEK2 kinase activity

**Figure 1:**
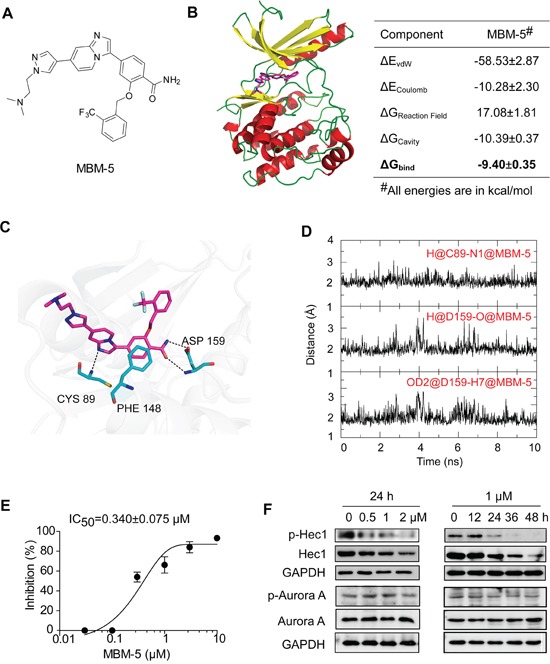
MBM-5 inhibits NEK2 kinase activity **A.** Chemical structure of MBM-5. **B-D.** Computational predicted binding mode of MBM-5 with NEK2. **B.** Bind free energies of MBM-5 to NEK2 calculated by the SIE method. ΔG_bind_ is the calculated binding free energy; ΔE_vdW_ and ΔE_Coulomb_ are contributions of van der Waals and Coulomb interactions to ΔG_bind_, ΔG_Reaction Field_ and ΔG_Cavity_ describe differences in the reaction field energy and molecular surface area upon inhibitor binding. C. Proposed binding mode of MBM-5 with NEK2 in the last snapshot of 20 ns molecular dynamic simulations. D. Time evolution of hydrogen bond distance between MBM-5 and NEK2 during MD simulations in the last 10 ns. **E.** The HTRF^®^ KinEASE™-STK kit was used to measure inhibition of NEK2 by MBM-5. The inhibition rates were determined in reference to the control. Data presented are the mean ± SD of three independent experiments. **F.** Effects of MBM-5 on phosphorylation of Hec1 and Aurora A in MGC-803 cells. The results are representative of three independent experiments.

MBM-5 is a novel compound synthesized in our laboratory (Figure 1A). The molecular docking/dynamic simulation combined with binding free energy calculations were used to study the binding modes of MBM-5 with NEK2. The predicted binding mode of MBM-5 and NEK2 showed that MBM-5 occupied the ATP-binding site and formed three conserved hydrogen bonds with NEK2 (Figure 1B-1D), one was between nitrogen atom of imidazo[1,2-a]pyridine and CYS 89, and the other two were between the amide group and ASP 159. In addition, there was a conserved π-π interaction between MBM-5 and NEK2. The calculated binding free energy between MBM-5 and NEK2 was −9.40 kcal/mol. The HTRF^®^ (Homogeneous Time-Resolved Fluorescence) KinEASE^™^-STK assay was then carried out to determine the NEK2 kinase inhibition activity of MBM-5. The result showed that the IC_50_ was 0.340±0.075 μM (Figure 1E). We further determined the inhibitory effect of MBM-5 on NEK2 kinase activity in cancer cells. As an important substrate of NEK2, Hec1 was phosphorylated by NEK2 at S165; therefore the phosphorylation of Hec1 (S165) was determined. MBM-5 effectively inhibited the phosphorylation of Hec1 (S165) in a concentration- and time- dependent manner, indicating MBM-5 was able to enter into cells and inhibit cellular NEK2 kinase activity (Figure 1F). Unexpectedly, the total Hec1 was down-regulated after NEK2 treatment. Considering the similar structure between NEK2 and Aurora A (with 31% structurally identical) [25], we determined the effect of MBM-5 against Aurora A kinase activity by analyzing its phosphorylated protein level. The phosphorylation of Aurora A was not affected by MBM-5 (Figure 1F). These data demonstrated that MBM-5 was able to inhibit NEK2 kinase activity.

### MBM-5 shows *in vitro* antitumor effects in a variety of cancer cells

**Figure 2:**
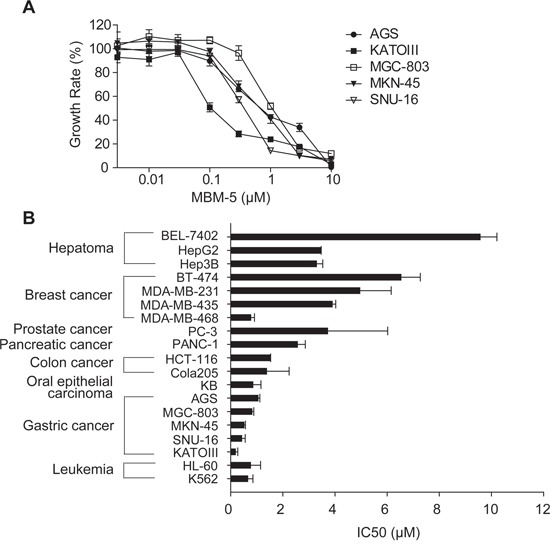
MBM-5 has *in vitro* antitumor activities against cancer cells **A** and **B.** Growth inhibition assays of MBM-5 *in vitro* were performed using the MTT assay. The growth rates were determined in reference to the control. The concentration–growth rate curves of MBM-5 on gastric cancer cells were drawn (A), and the IC_50_ was calculated (B). Data presented are the mean ± SD of three independent experiments.

Because the NEK2 kinase is essential for cell proliferation, we examined the anti-proliferative effects of MBM-5 on a panel of 19 cancer cell lines, including leukemia, gastric, colorectal, prostate, breast and hepatoma cancer cells. Representative concentration-inhibition curves were drawn as shown in Figure 2A, and IC_50_ values were calculated and listed in Figure 2B and Table 1. The IC_50_ values ranged from 1 to 10 μM and leukemia, gastric and colorectal cancer cell lines were relatively sensitive to MBM-5 than other cell lines. These data were consistent with the prediction that NEK2 kinase activity is crucial for cellular proliferation, and in line with the findings that NEK2 depletion inhibits cancer cell growth.

**Table 1:** IC_50_ values for inhibition of cell growth by MBM-5 measured via MTT assay

Cell Type	Cell line	IC_50_ (μM)
Chronic myeloid leukemia	K562	0.65±0.20
Acute myeloid leukemia	HL-60	0.76±0.38
Gastric cancer	KATOIII	0.18±0.09
	SNU-16	0.42±0.13
	MKN-45	0.51±0.05
	MGC-803	0.81±0.07
	AGS	1.05±0.07
Oral epithelial carcinoma	KB	0.85±0.31
Colorectal cancer	Cola205	1.38±0.87
	HCT-116	1.51±0.02
Pancreatic cancer	PANC-1	2.56±0.30
Prostate cancer	PC-3	3.72±2.30
Breast cancer	MDA-MB-468	0.77±0.14
	MDA-MB-435	3.89±0.14
	MDA-MB-231	4.95±1.20
	BT-474	6.53±0.74
Hepatoma	Hep3B	3.29±0.24
	HepG2	3.44±0.03
	BEL-7402	9.57±0.64

### MBM-5 induces chromosomal misalignment and triggers mitotic catastrophe

**Figure 3:**
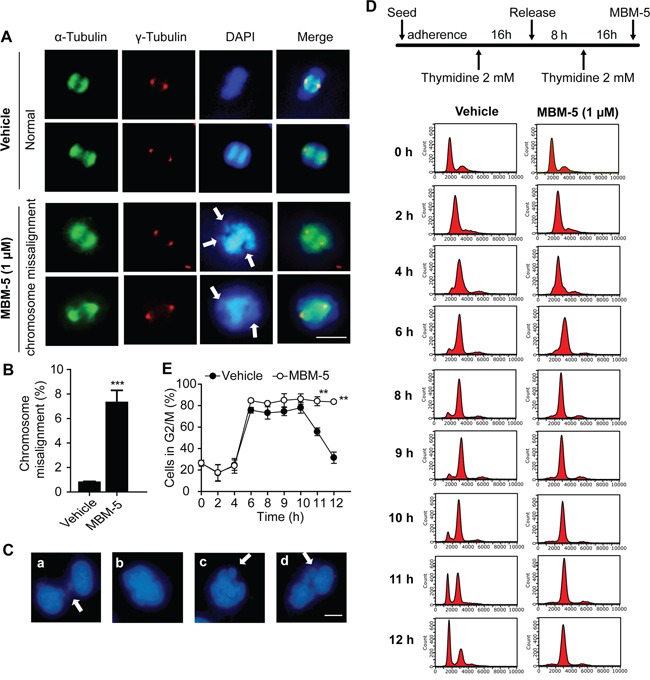
MBM-5 triggers mitotic catastrophe **A.** Representative images and quantitation of misaligned metaphase chromosomes in MGC-803 cells treated with DMSO and 1 μM of MBM-5 for 12 h. Centrosome, microtubules and chromosome were stained with anti-γ-tubulin, anti-α-tubulin and DAPI, respectively. Scale bar, 10 μm. **B.** Cells with misaligned metaphase chromosomes in total cells were quantitated. Error bar represent SD, n≥200 per sample. **C.** Representative images of mitotic progression defects in MGC-803 cells treated with 1 μM of MBM-5 for 12 h. (a) thin chromatin bridges, (b) binucleated cells, (c and d) micronucleated cells. Scale bar, 10 μm. **D.** Schematic of the protocol used to treat the cells. Following DTB, MGC-803 cells were released into fresh medium in the presence of DMSO or MBM-5 (1 μM). DNA content of cells collected at the indicated time points was assessed by flow cytometric analysis of cells labeled with propidium iodide. The results are representative of three independent experiments. **E.** The percentages of G2/M phase cells in (D) were quantitated. Data presented are the mean ± SD of three independent experiments.

Perturbation of NEK2 function by RNAi or overexpression of kinase-inactive NEK2 leads to mitotic abnormalities represented by spindle configuration changes and chromosome misalignment. We then detected whether MBM-5 treatment would elicit these phenotypes. In contrast to DMSO treatment, MGC-803 cells displayed increased chromosomal misalignment after treated with MBM-5 for 12 h (Figure 3A). The proportion of cells with chromosomal misalignment increased from 0.79% in control group to 7.30% in MBM-5 treated cells (Figure 3B). Multipolar spindle configurations in the mitotic population could be observed, albeit with a very small population (Data not shown). Mitotic progression defects were further evidenced by the appearance of thin chromatin bridges, binucleated and micronucleated interphase cells (Figure 3C).

To determine whether these mitotic defects would lead to cell cycle arrest, cell cycle analysis was performed. MGC-803 cells were first synchronized in G1/S phase by double thymidine block (DTB) treatment, and then were released into MBM-5 in order to better understand the effect of MBM-5 on cell cycle progression. As shown in Figure 3D and 3E, both control and MBM-5 treated cells progressed to G2/M phase by 6 h after DTB release. For control cells, about 80% of cells remained in G2/M phase by 10 h and then gradually progressed from G2/M to G1 phase at 11-12 h. In contrast, cells treated with MBM-5 were still arrested in G2/M phase at least 12 h after DTB release. Together, these observations indicated that MBM-5 induced mitotic catastrophe by inducing chromosomal misalignment and cytokinesis failure, without affecting mitotic entry.

### MBM-5 induces cell cycle arrest and polyploid

**Figure 4:**
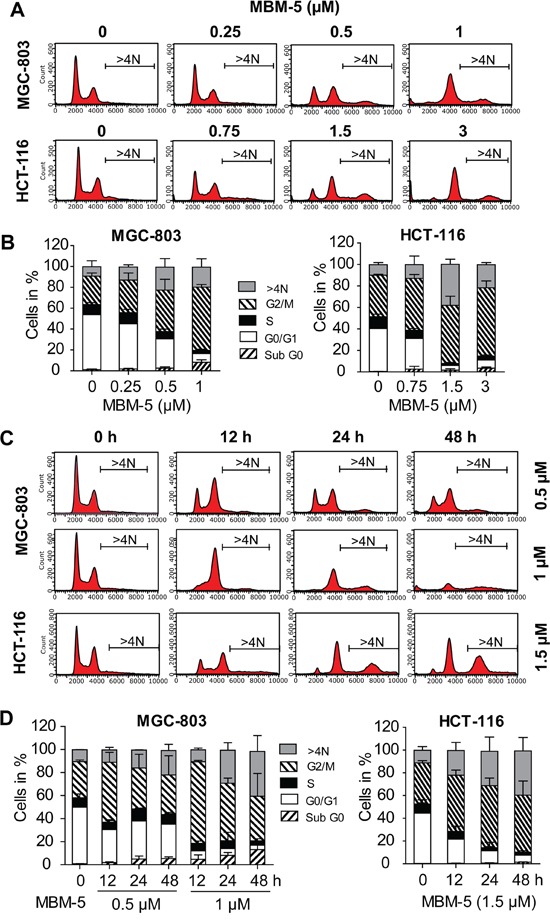
MBM-5 causes accumulation of cells with ≥4N DNA content **A.** MGC-803 and HCT-116 cells were treated with indicated concentration of MBM-5 for 24 h. Cell cycle distribution was assessed by flow cytometric analysis of cells labeled with propidium iodide. Representative data were shown. **B.** Cell cycle proportion in (A) was quantitated. Data presented are the mean ± SD of three independent experiments. **C.** MGC-803 and HCT-116 cells were treated with indicated concentration of MBM-5 for different times. Cell cycle distribution was assessed by flow cytometric analysis of cells labeled with propidium iodide. Representative data were shown. **D.** Cell cycle proportion in (C) was quantitated. Data presented are the mean ± SD of three independent experiments.

To fully understand the effect of MBM-5 on mitotic progression, asynchronously-growing MGC-803 and HCT-116 cells were treated with serial dilutions of MBM-5 or with different times. In both cell lines, rising concentrations caused a pronounced enrichment of G2/M phase cells (Figure 4A and 4B). For example, the population of MGC-803 cells at G2/M phase increased from 27.2% to 31.2%, 39.7% and 60.6% after treated with 0.25, 0.5 and 1 μM MBM-5 for 24 h, respectively. Interestingly, an increased population of cells with >4N DNA content was observed after MBM-5 treatment, which was associated with a repeated round of DNA synthesis after cytokinesis failure. We then determined the cell cycle progression after MBM-5 treatment over time. In MGC-803 cells, the population of cells at G2/M phase reached peak level after treated with MBM-5 (0.5 or 1 μM) for 12 h, and then decreased gradually at 24 h and 48 h. Meanwhile, the sub G0 and >4N population increased in a time-dependent manner, suggesting cells arrested in 4N either underwent cell death or entered the G1 phase without cell division. Similar phenomenon was also observed in HCT-116 cells (Figure 4C and 4D).

**Figure 5:**
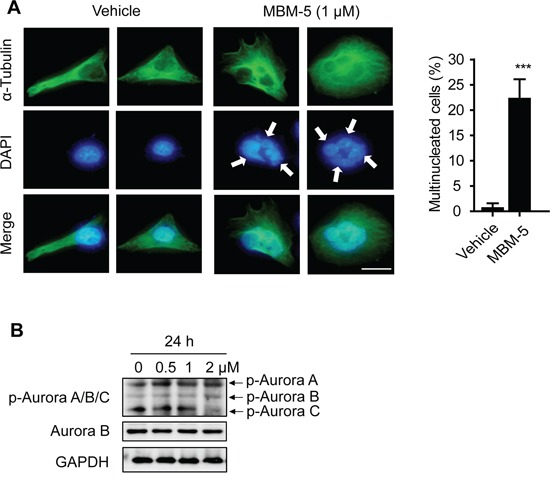
MBM-5 increases formation of multinuclear cells **A.** Representative images and quantitation of multinuclear MGC-803 cells treated with DMSO and 1 μM of MBM-5 for 24 h. Microtubules and chromosome were stained with anti-α-tubulin and DAPI, respectively. Data show percentage of cells with multinucleation in total cells. Error bar represent SD, n≥200 per sample. Scale bar, 20 μm. **B.** Effects of MBM-5 on phosphorylation of Aurora A/B/C kinases in MGC-803 cells treated with MBM-5. The results are representative of three independent experiments.

In line with the above results, microscopic examination revealed that some cells underwent chromosome decondensation and formation of nuclear envelopes without the normal processes of mitosis. Therefore, parts of cells with 4N DNA content were actually arrested in pseudo-G1 phase with binuclear (Figure 3C). With a repeated round of DNA synthesis in a subsequent S phase, these cells became containing 8N DNA content (Figure 4). In consistence, the multinucleated cells increased from 0.6% to 22.2% after treated with MBM-5 (1 μM) for 24 h, further confirming the perturbed mitotic progression (Figure 5A).

Inhibition of Aurora B kinase activity was reported to cause polyploidy; therefore we speculated whether accumulated cells with >4N DNA content were resulted from inhibition of Aurora B kinase by MBM-5. In our study, we used an antibody that recognizes phospho-Aurora A (T288)/Aurora B (T232)/Aurora C (T198) to determine the activation of Aurora kinases. MBM-5 treatment did not inhibit the phosphorylation of Aurora A/B/C, ruling out all these three kinases as direct targets of MBM-5 (Figure 5B).

### MBM-5 induces apoptosis

**Figure 6:**
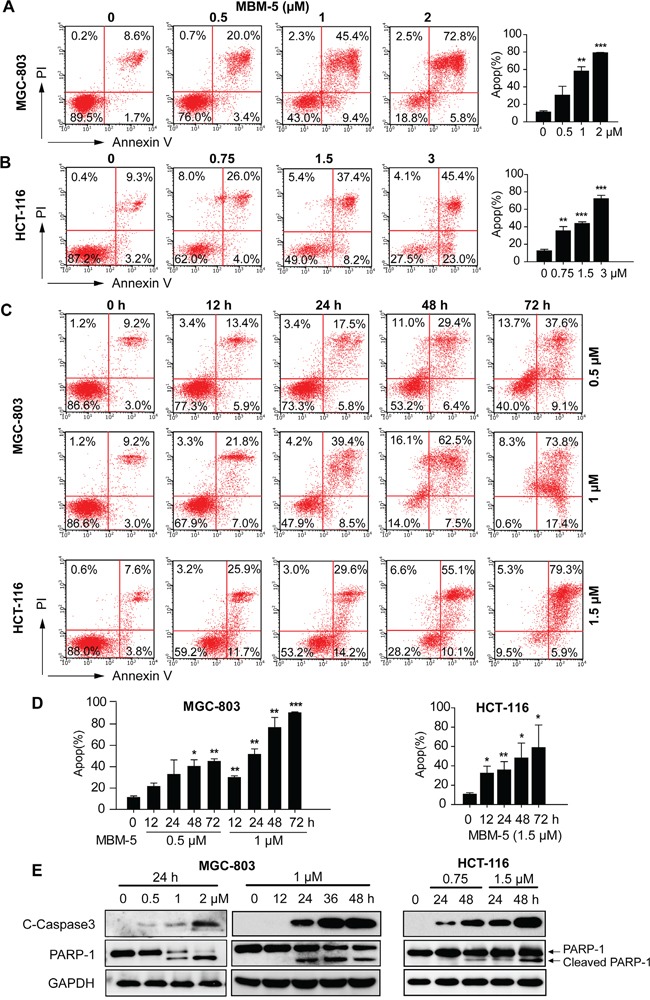
MBM-5 induces apoptosis **A** and **B.** MGC-803 and HCT-116 cells were treated with indicated concentration of MBM-5 for 24 h. **C** and **D.** MGC-803 and HCT-116 cells were treated with indicated concentration of MBM-5 for different times. Apoptosis was assessed by flow cytometric analysis after Annexin V/PI staining. Representative images and quantitation of apoptotic cells were shown. Data presented are the mean ± SD of three independent experiments. **E.** Effects of MBM-5 on protein expression of cleaved caspase 3 and PARP-1. The results are representative of three independent experiments.

Given the increased sub G0 population after MBM-5 treatment over time (Figure 4), we then determined whether MBM-5 treatment led to apoptosis or necrosis by performing flow cytometry with Annexin-V/PI staining. As shown in Figure 6A-6D, MBM-5 induced apoptosis of MGC-803 and HCT-116 cells, both in a concentration- and time-dependent manner. For example, the percentage of apoptotic HCT-116 cells increased to 30.0%, 45.6% and 68.4%, after treated with 0.75, 1.5 and 3 μM MBM-5 for 24 h, respectively. Moreover, the apoptotic population reached 91.2% and 85.2% after treated with 1 μM and 1.5 μM MBM-5 for 72 h in MGC-803 and HCT-116 cells, respectively. Western blot results showed that MBM-5 treatment resulted in the cleavage of caspase 3 and PARP-1, indicating that MBM-5 activated the apoptotic pathway (Figure 6E).

### MBM-5 effectively suppresses tumor growth in human gastric and colorectal cancer cells xenografts

**Figure 7:**
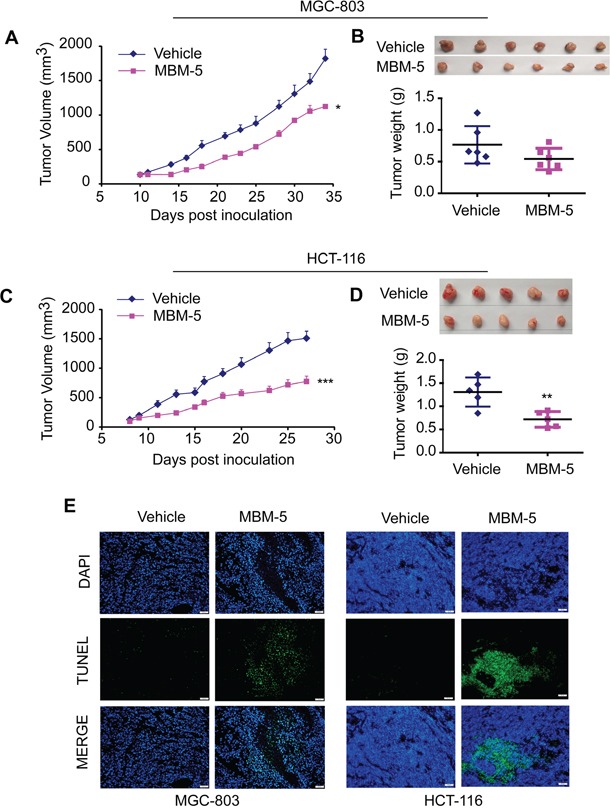
MBM-5 retards tumor growth *in vivo* MGC-803 and HCT-116 xenograft bearing nude mice were treated with 30 mg/kg and 20 mg/kg MBM-5 as indicated schedule, respectively. **A** and **C.** The tumor volume was recorded and plotted against days post inoculation. Data presented are the mean ± SEM (n=6 in MGC-803 xenograft model, n=5 in HCT-116 xenograft model). **B** and **D.** The pictures of tumors and tumor weights. **E.** Representative images derived from TUNEL staining to evaluate the apoptosis in tumor tissues. Scale bar, 50 μm.

To determine the antitumor effect of MBM-5 *in vivo*, we established a subcutaneous gastric carcinoma model using MGC-803 cells and colorectal cancer model using HCT-116 cells. To improve the water solubility of compound MBM-5, the succinic acid salt of MBM-5 was synthesized for animal studies. And the salt form of MBM-5 was confirmed to have the same anti-cancer activity *in vitro* (Data not shown). For gastric carcinoma model, tumor growth was suppressed after MBM-5 (30 mg/kg) treatment when compared with vehicle-treated mice. At the end of the experiment, the tumor volume was significantly reduced (*p*<0.05) with a TGI% of 42.4% (Figure 7A). The average tumor weight of MBM-5-treated mice was 0.54±0.17 g, which was virtually lower than vehicle-treated mice (0.77±0.29 g), albeit without significant difference (Figure 7B). In colorectal cancer model, mice received 20 mg/kg of MBM-5 showed a significant reduction in tumor volume commencing on day 4. At the end of the experiment, the tumor volume was significantly inhibited (*p*<0.001) compared with control, with a TGI% of 51.1% (Figure 7C). Moreover, the average tumor weight of MBM-5-treated mice was 0.72±0.17 g, which was significantly lower (*p*<0.01) than vehicle-treated mice (1.31±0.31 g) (Figure 7D). Throughout the experiments, mice treated with MBM-5 displayed neither drug-related death nor remarkable body weight loss of more than 20% (Data not shown).

Tumor tissues were assayed for apoptosis using a TUNEL kit which labels apoptotic nuclei with a fluorescent dye. As shown in Figure 7E, tumor tissues from animal receiving MBM-5 treatment showed an obvious increase in apoptosis. These findings coincided with the apoptotic effect of MBM-5 *in vitro*, highlighting the involvement of apoptosis in the tumor growth inhibitory effects exerted by MBM-5 *in vivo*.

## DISCUSSION

Elevated expression of NEK2 appears to participate in the initiation, maintenance, progression, metastasis of cancer and is positively associated with poor prognosis. RNAi depletion of NEK2 effectively inhibited proliferation of cancer cells and tumor growth in xenograft model [18, 19]. Moreover, compounds (INHs and TAIs) that specifically disrupted the Hec1/NEK2 interaction via direct Hec1 binding and caused NEK2 degradation significantly suppressed tumor growth *in vivo* without obvious toxicity [19]. Therefore, NEK2 is a promising therapeutic target for cancer treatment. However, the development of NEK2 inhibitors was at the very beginning, as the current reported NEK2 inhibitors was either with low activity or lack of membrane permeability. Great efforts should be made to develop NEK2 inhibitors that are suitable for clinical studies.

In this study, our library's compound MBM-5 was presumed to have potent activity against NEK2 kinase by docking simulation study. The results of the NEK2 kinase assay clearly showed that MBM-5 potently and concentration dependently inhibited NEK2 kinase activity *in vitro*. We further found that MBM-5 was able to inhibit the cellular kinase activity of NEK2 as evidenced by the decreased phosphorylation of Hec1 (S165). Although the protein level of total Hec1 was down-regulated by MBM-5, it seems not contribute to the decreased phosphorylation of Hec1. Because the phosphorylation of Hec1 was almost undetectable after treated with MBM-5 (0.5 or 1 μM) for 24 h, meanwhile a modest decrease of total Hec1 was observed. A previous study has reported similar phenomenon that the decreased phosphorylation of Hec1 was followed with a down-regulation of Hec1 [32]. Therefore, the phosphorylation of Hec1 might be correlated with the protein level, either affecting its transcription or post-translation modification, which will be studied in our further research. Moreover, MBM-5 prevented proliferation of a diverse range of human cancer cell lines with IC_50_ values of micromole range. The inhibition was associated with accumulation of G2/M cells, pseudo-G1 cells, and induction of polyploidy cells, as well as apoptosis.

Perturbation of NEK2 function by RNAi or overexpression of kinase-inactive NEK2 was found to cause multipolar spindle configurations [23, 24, 33]. Inhibitors that disrupt NEK2/Hec1 interaction led to aberrant spindles, such as deformed or multipolar spindles [32, 34]. However, this observation was challenged by a study also based on NEK2 RNAi. It reported that NEK2 depleted cells were capable of forming normal bipolar spindles with fully active kinesin motor Eg5 [35]. Therefore, the exact role of NEK2 in bipolar spindle assembly remains controversial. In our experiment, about 90% of mitotic cells exhibited normal bipolar spindle after MBM-5 treatment, suggesting NEK2 might play a supporting role rather than an indispensible role in bipolar spindle assembly.

The spindle assembly checkpoint (SAC) delays sister chromatid separation until all chromosomes are attached to the bipolar spindle. It is able to detect a single unaligned chromosome, causing a prolonged mitotic arrest [36]. Phosphorylation of Hec1 (S165) by NEK2 modulates chromosome alignment and serves as an intricate molecular switch modulating the activity of the SAC. Lack of Hec1 (S165) phosphorylation weakens the SAC signaling and triggers onset of anaphase even with unaligned chromosomes [9]. Here we observed that MBM-5 effectively inhibited the phosphorylation of Hec1 (S165) by NEK2, thus leading to chromosome segregation defects represented by misaligned chromosomes in metaphase cells and thin chromatin bridges in telophase cells.

In both synchronized and asynchronously-growing cells, MBM-5 treatment induced an obvious accumulation of cells with 4N DNA content. Some of these cells were actually in pseudo-G1 phase, as evidenced by the observation of binuclear cells. These might be resulted from mitotic slippage, which means cells escape from mitotic arrest and subsequently enter the G1 phase without cell division. In addition, MBM-5 also increased the proportion of cells with >4N DNA content. Generally speaking, the generation of cells with >4N DNA content could occur by two distinct mechanisms. Cells replicate their DNA without entering mitosis after completion of S phase; or cells proceed through mitosis without dividing, and replicate their DNA in a subsequent S phase [37]. In our experiment, MGC-803 cells released from a G1/S block was able to progress into G2/M phase after treated with MBM-5 for 6 h, suggesting that cells were able to enter mitosis. Therefore MBM-5-induced accumulation of population with >4N DNA content represented cells that exited mitosis without cell division and subsequently experienced a repeated round of DNA synthesis. As a result, MBM-5 treatment increased the level of multinucleated cells. Our study was in consistent with a previous study that some NEK2B depleted cells were unable to complete cytokinesis resulting in the formation of multinucleated cells [11]. Therefore, our study provided a new evidence that NEK2 might regulate cytokinesis.

Cells with prolonged mitotic arrest or continued proliferation with cytokinesis failure subsequently underwent apoptosis. In both MGC-803 and HCT-116 cells, the predominant effect of MBM-5 at 12 h was the accumulation of cells with 4N DNA content. At 24 h, the repeated round of DNA synthesis (represented as >4N DNA content population) occurred in 10-30% cells, and then increased slightly at 48 h. However, no further increase in the degree of polyploidy was observed up to 96 h (Data not shown). Meanwhile, the apoptosis increased significantly with time, indicating MBM-5 triggered apoptosis either during mitotic arrest or after mitotic slippage to a new round of mitotic progression. Moreover, MBM-5 induced apoptosis was also observed *in vivo* as evidenced by the increased apoptotic nuclei in MBM-5 treated tumor tissues.

Before evaluating the anti-tumor effect of MBM-5 *in vivo*, we first determined the pharmacokinetic parameters by single i.v. (2 mg/kg) administration of MBM-5 on rats. Using the MBM-5 plasma concentration-time profile curve (Supplementary data 1, Supplementary Figure 1), the pharmacokinetic parameters were determined using Noncompartmental Analysis. The plasma concentration was cleared with a half-life (t_1/2_) of 1.5 h and clearance (CL) rate of 121 ml/h/kg (Supplementary data 1, Supplementary Table 1). Keeping this in mind, we dosed xenograft mice twice a day. Compared with vehicle treated mice, MBM-5 treatment retarded the tumor growth in both gastric and colorectal cancer cells xenograft models. However, tumor stasis or regression was not observed. Due to the short half-life, more frequent dosing may be needed to enhance the antitumor activity *in vivo*. Anyway, MBM-5 was the first ATP-competitive NEK2 inhibitor that exhibited the antitumor effect both *in vitro* and *in vivo*, therefore was an ideal lead compound worth for further optimization. To improve its kinase inhibitory activity and pharmacokinetic parameters, further chemical modification of MBM-5 is currently in progress.

In conclusion, MBM-5 was identified to be one of the most promising lead compounds of ATP-competitive NEK2 inhibitors. It demonstrated prominent *in vitro* antitumor activity against a wide variety of cancer cells. MBM-5 interrupted mitotic progression by inducing chromosome segregation defects and cytokinesis failure; therefore leading to accumulation of cells with 4N DNA content (G2/M phase or binuclear) and >4N DNA content (polyploidy or multinuclear), which finally underwent apoptosis. The antitumor potential was further strengthened by its suppressive effect on the growth of MGC-803 gastric and HCT-116 colorectal tumor xenografts *in vivo*, even though its half-life *in vivo* was quite short. Together, MBM-5 has been established unequivocally as a lead compound by targeting NEK2 kinase in this study.

## MATERIALS AND METHODS

### Compounds

MBM-5 (4-(7-(1-(2-(dimethylamino)ethyl)-1H- pyrazol-4-yl)imidazo[1,2-a]pyridin-3-yl)-2-((2-(trifluoromethyl)benzyl)oxy)benzamide), with purity>98% was synthesized by our group. MBM-5 was dissolved in DMSO as 30 mM stock solution for enzymatic assays and *in vitro* cell based assays. The succinic acid salt of MBM-5, with purity>98% was synthesized and dissolved in ultrapure water for animal studies; the solution was freshly prepared before each use. The information about the purities of MBM-5 and its salt was shown in Supplementary data.

### Cell culture and synchronization

All cell lines were purchased from Cell Bank of China Science Academy (Shanghai, China) and maintained in growth medium as recommended at 37°C in a 5% CO2 atmosphere. Penicillin (100 U/ml) and streptomycin (100 μg/ml) were added in the medium. Double thymidine block (DTB) was performed as previously described [38].

### Animal treatment

Female BALB/c nu/nu mice (5-6 weeks, 16-18 g) were obtained from Sino-British SIPPR/BK Lab. Animal Ltd, Shanghai, China, with the certification number of 2008001638201. The animals were housed in specific pathogen-free (SPF) conditions at Key Laboratory of Brain Functional Genomics, Ministry of Education, East China Normal University, and were acclimatized for a week prior to use. The use and care of experimental animal was approved by Institutional Animal Care and Use Committee, East China Normal University.

### Molecular modeling

X-ray protein structure of NEK2 (PDB ID: 4AFE) was obtained in Protein Data Bank. All water molecules in the crystal structure were removed, and then the structure was used for molecular docking using Sybyl X2. For docked structure, energy minimization and molecular dynamic (MD) simulation were performed using the Sander module in Amber14. A two-step, extensive energy minimization process based on the steepest descent method followed by the conjugate gradient algorithm were carried out to relieve bad contacts and to direct each system toward energetically favorable conformations. After minimization, each system was gently heated from 0 to 300 K in 500 ps at constant volume and equilibrated at 300 K for another 500 ps. Finally, a 20 ns MD simulation without any restrictions was performed at constant pressure, and the coordinates of atoms were saved every 2 ps. During the MD simulation, all bonds involving hydrogen atoms were constrained using the SHAKE algorithm, and a time step of 2 fs was adopted. The temperature was controlled using the Langevin thermostat with a collision frequency of 2.0 ps−1. The particle mesh Ewald (PME) method was applied to treat the long-range electrostatic interactions. The cutoff distances for the long-range electrostatic and van der Waals interactions were set to 12.0 Å. Solvated interaction energy (SIE) method was used to calculate the binding free energies between compounds and NEK2. For the current work, 200 snapshots extracted from the last 10 ns of MD trajectory at an interval of 50 ps were used for the binding free energy analyses.

### Kinase assays

The inhibition of MBM-5 on activity of NEK2 kinase (Millipore) was characterized by HTRF^®^ KinEASE™-STK kit according to the manufacturer's instructions (Cisbio Assays).

### Cell proliferation assay

Cell proliferation was assessed by the MTT assay [39]. The inhibition rate for each well was calculated as (ODcontrol cells−ODtreated cells)/ODcontrol cells × 100%. The IC_50_ values were calculated by concentration−response curve fitting using the four parameter method.

### Cell cycle analysis

Cell cycle distribution was determined by flow cytometry measurement of DNA content after cells were incubated with RNase A (10 mg/ml) and propidium iodide (50 mg/ml). The cellular DNA content was analyzed on a flow cytometer (Guava EasyCyte 6HT2L). The percentage of each population was measured using the InCyte software. At least 20 000 cells were analyzed for each data point.

### Apoptosis analysis

Apoptosis was detected by FITC Annexin V Apoptosis Detection Kit I according to the manufacturer's instructions (Dojindo) to determine the phosphatidyl serine exposure. Apoptotic cells were quantified by a flow cytometer (Guava EasyCyte 6HT2L) using the InCyte software. At least 10 000 cells were analyzed for each data point.

### Immunoblotting analysis

Immunoblotting analysis of proteins in cell lysates was performed as previously described [40]. The expression of GAPDH was used as loading control. Primary antibodies used were as follows: anti-NEK2 (#610593) was purchased from BD; anti-p-Hec1(Ser165) (#GTX70013) and anti-Hec1 (#GTX70268) were purchased from Gene Tex; anti-p-Aurora A (#3079), anti-Aurora A (#12100), anti-p-Aurora A/Aurora B/Aurora C (#2914), anti-Aurora B (#3094), and anti-cleaved caspase 3 (#9661) were purchased from Cell Signaling Technology; anti-PARP (#sc-8007) and anti-GAPDH (#sc-25778) were purchased from Santa Cruz.

### Immunofluorescence

Cells were grown on 96-well tissue culture plate (Corning, NY) and permeabilized with 0.5% Triton-X100 in PHEM Buffer (80 mM PIPES, 25 mM HEPES, pH 7.2, 10 mM EGTA, 4 mM MgSO_4_) for 10 min at room temperature, then fixed in PHEM buffer containing 4% paraformaldehyde for 20 min. Cells were blocked with 3% BSA plus 10% FBS in PHEM, and then incubated with primary antibodies: anti-α-tubulin (TU-02) (Santa Cruz) and anti-γ-tubulin (Sigma) in PBS overnight at 4°C. The secondary antibodies Alexa Fluor^®^ 488 goat anti-rabbit IgG (H+L) or Alexa Fluor^®^ 594 goat anti-mouse IgG (H+L) (Invitrogen) were applied respectively for two hours after the plate were washed with TBS thoroughly. 4′, 6-Diamidino-2-phenylindole (DAPI) staining was applied after secondary antibody incubation. Images were captured with a fluorescence microscopy (Olympus IX3).

### Antitumor activity *in vivo*

Human MGC-803 xenografts and HCT-116 xenografts were established by subcutaneously inoculating 5 × 10^6^ cells into nude mice. When the tumors reached a mean group size of 100~150 mm^3^, the mice were randomized into control and treatment groups to receive treatment accordingly. Mice were treated with vehicle or MBM-5 twice a day via intraperitoneal injection. The tumor growth was recorded with the measurement of length (L) and width (W) by caliper three times a week, and calculated as tumor volume (V) = L × W^2^/2. The tumor growth inhibition was calculated as TGI%= (1-(mean tumor volume of the treatment group on the first day-mean tumor volume of the treatment group on the end day)/(mean tumor volume of the control group on the first day-mean tumor volume of the control group on the end day)) x100%.

### TUNEL histology

To evaluate the apoptotic response in tumor tissue, we applied terminal deoxynucleotidyl transferase (TdT)-mediated dUTP-digoxigenin nick-end labeling technique (TUNEL), to formalin-fixed tumor samples in paraffin blocks, using the commercially available Kit (Promega). The sections (4–5 μm) mounted on glass slides were deparaffinized, rehydrated through graded alcohols to water, treated with 20 μg/ml proteinase K (37°C, 20 min) and then washed in 1× Tris buffer. TUNEL assay was then performed according to the instructions by the manufacturer. Images were captured with a fluorescence microscopy (Olympus IX3).

### Statistical analysis

The statistical significance of differences between groups was evaluated by the unpaired Student's t test and indicated with ***P<001, **P<0.01, *P<0.05. All statistical tests were two sided.

## SUPPLEMENTARY DATA



## Abbreviations

DTB: double thymidine block
Hec1: highly expressed in cancer 1
HTRF: Homogeneous Time-Resolved Fluorescence
MAD1: mitotic arrest deficient-like 1
MD: molecular dynamic
NEK2: Never in Mitosis (NIMA) Related Kinase 2
NEKs: Never in Mitosis (NIMA) Related Kinases
Nlp: ninein-like protein
PME: particle mesh Ewald
SAC: spindle assembly checkpoint
SIE: solvated interaction energy
SPF: specific pathogen-free
TUNEL: terminal deoxynucleotidyl transferase (TdT)-mediated dUTP-digoxigenin nick-end labeling technique
