# Supplementary material for: Preclinical activity of MBM-5 in gastrointestinal cancer by inhibiting NEK2 kinase activity
Source: Oncotarget. 2016 Oct 15;7(48):79327–41. doi: 10.18632/oncotarget.12687 (PMC5346717; doi:10.18632/oncotarget.12687)
Supplement: Supplementary file 1 [file oncotarget-07-79327-s001.pdf]

Preclinical activity of MBM-5 in gastrointestinal cancer by inhibiting NEK2 kinase activity

SUPPLEMENTARY DATA

Pharmacokinetic Parameters Obtained in Rats. The succinic acid salt of MBM-5 were intravenously administrated to SD rats (n = 3) at 2 mg/kg. Blood samples were collected at 0.08, 0.25, 0.5, 1, 1.5, 2, 4, 8 and 24 h time points following intravenous dosing. The blood samples were placed on wet ice, and blood plasma was collected after centrifugation. Serum samples were frozen and stored at −80 °C. The serum samples were analyzed utilizing HPLC-coupled tandem mass spectrometry (LC−MS/MS). The pharmacokinetic data analysis was performed using noncompartmental analysis modules in WinNonlin Professional v6.3 (Pharsight, USA).

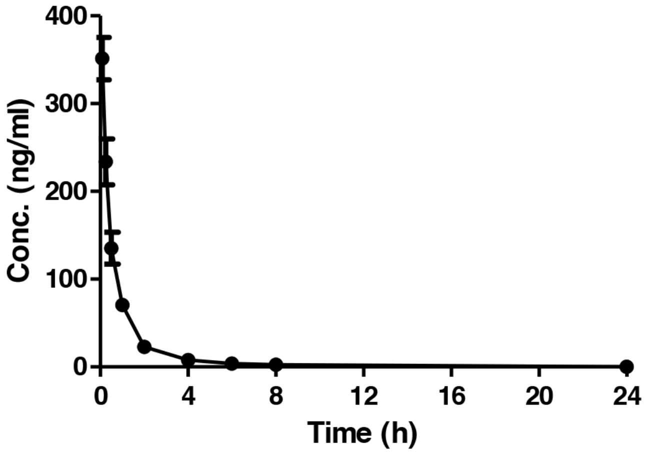

Supplementary Figure 1: MBM-5 plasma concentration-time profile in rats following single i.v. administration of 2 mg/kg MBM-5. Each point represents mean±SD of three rats.

MBM-5 (HPLC analysis: 98.86%)

Chromatogram

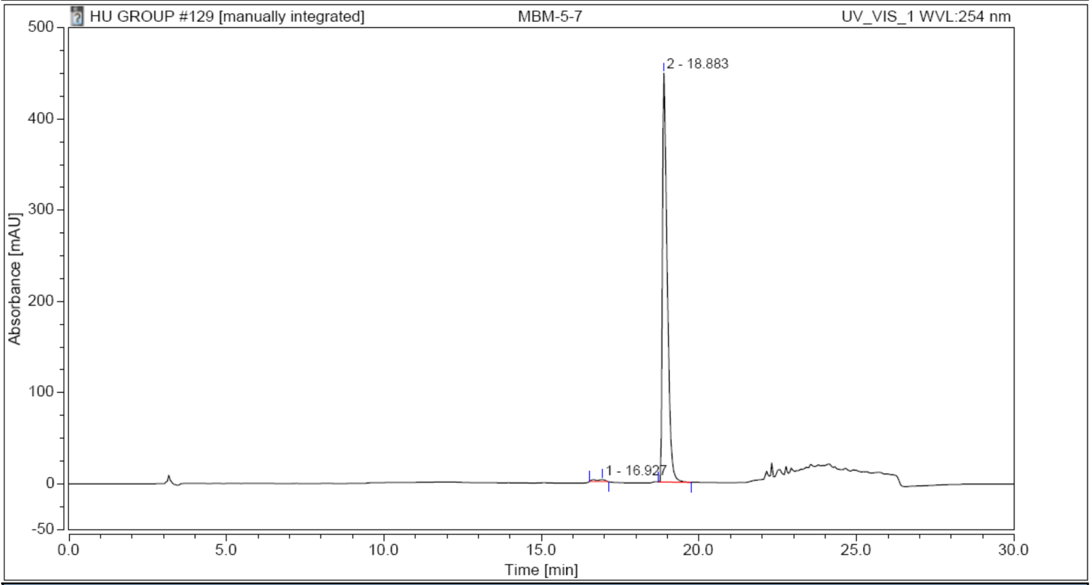

Integration Results

No.	Peak Name	Retention Time min	Area mAU*min	Height mAU	Relative Area %	Relative Height %	Amount n.a.
1		16.927	0.903	2.201	1.14	0.49	n.a.
2		18.883	78.539	448.323	98.86	99.51	n.a.
**Total:**			**79.441**	**450.524**	**100.00**	**100.00**	

MBM-5: ^{1}H NMR (400 MHz, MeOD) δ 8.28 (d, *J* = 7.2 Hz, 1H), 8.25 (s, 1H), 8.07 (d, *J* = 8.1 Hz, 1H), 7.97 (s, 1H), 7.78 (m, 2H), 7.72 – 7.62 (m, 3H), 7.56 (t, *J* = 7.6 Hz, 1H), 7.30 (dd, *J* = 8.1, 1.2 Hz, 1H), 7.23 (s, 1H), 7.13 (dd, *J* = 7.2, 1.4 Hz, 1H), 5.53 (s, 2H), 4.42 (t, *J* = 6.3 Hz, 2H), 3.15 (t, *J* = 6.3 Hz, 2H), 2.54 (s, 6H).

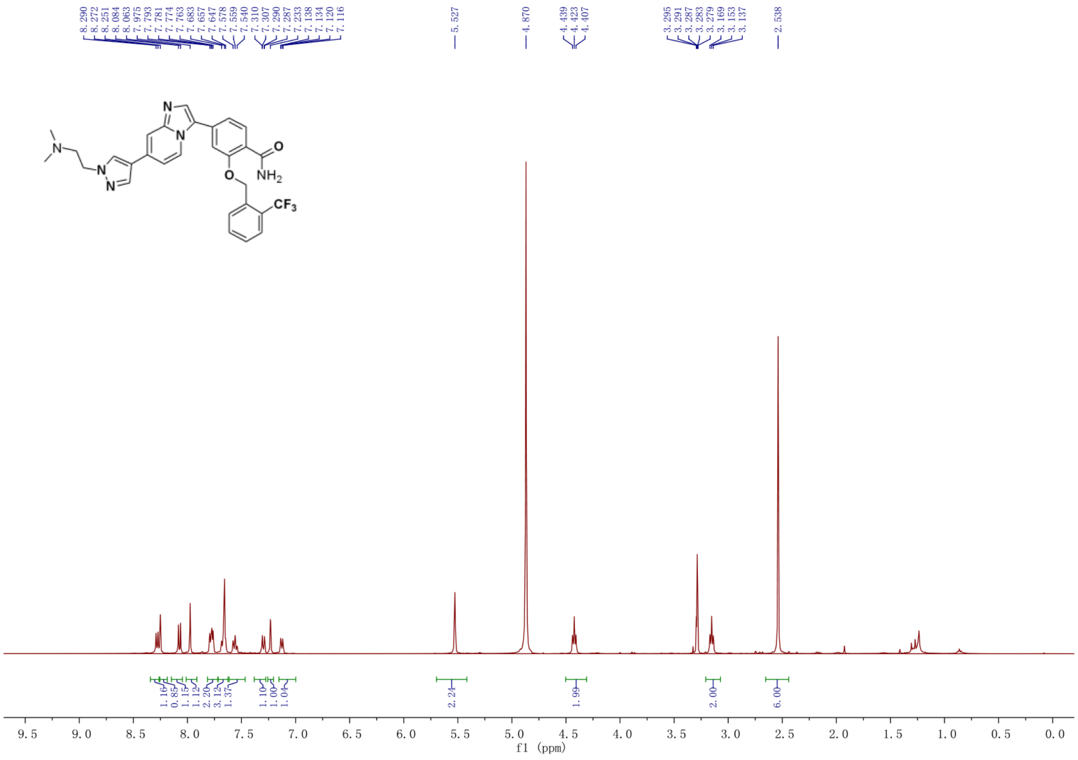

MBM-5: ^{13}C NMR (100 MHz, $CDCl_3$) δ 166.35, 157.02, 147.38, 136.97, 134.32, 133.99, 133.71, 133.66, 132.64, 129.90, 129.51, 128.87, 128.03 (q, J = 30.8 Hz), 127.40, 126.54 (q, J = 5.6 Hz), 124.38, 124.15 (q, J = 272.2 Hz), 123.38, 120.75, 120.61, 120.08, 112.31, 112.13, 111.52, 67.66, 58.68, 50.19, 45.37.

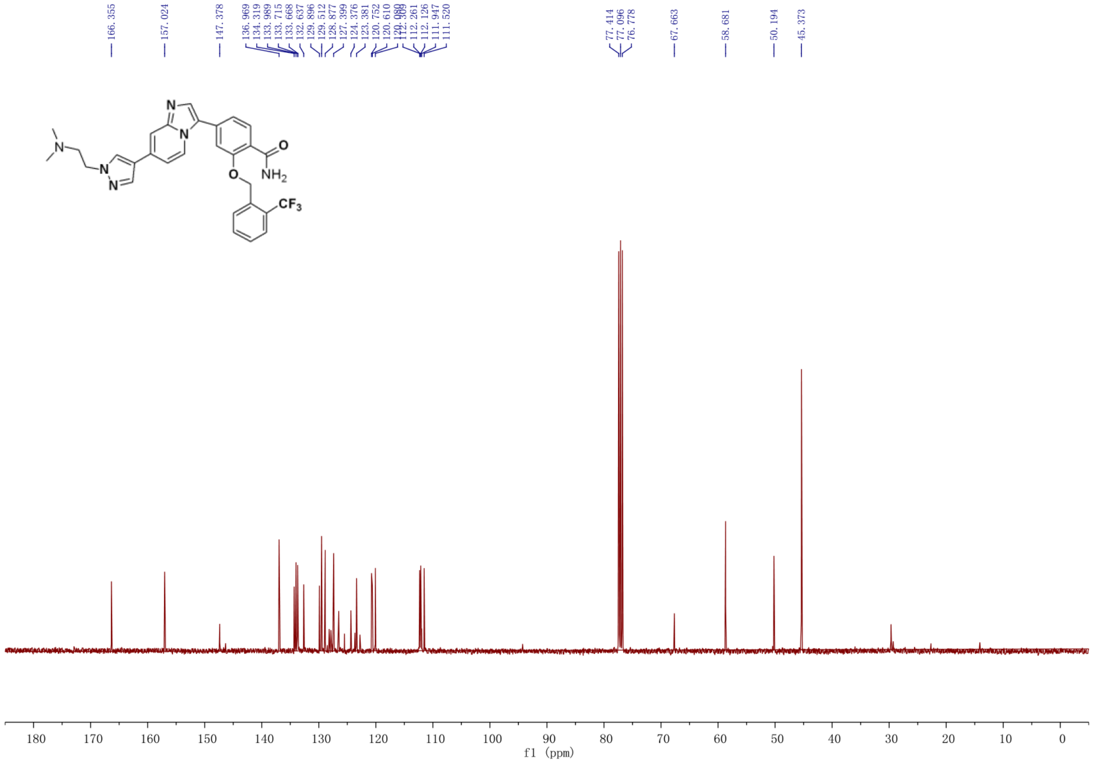

The succinic acid salt of MBM-5 (HPLC analysis: 99.02%)

Chromatogram

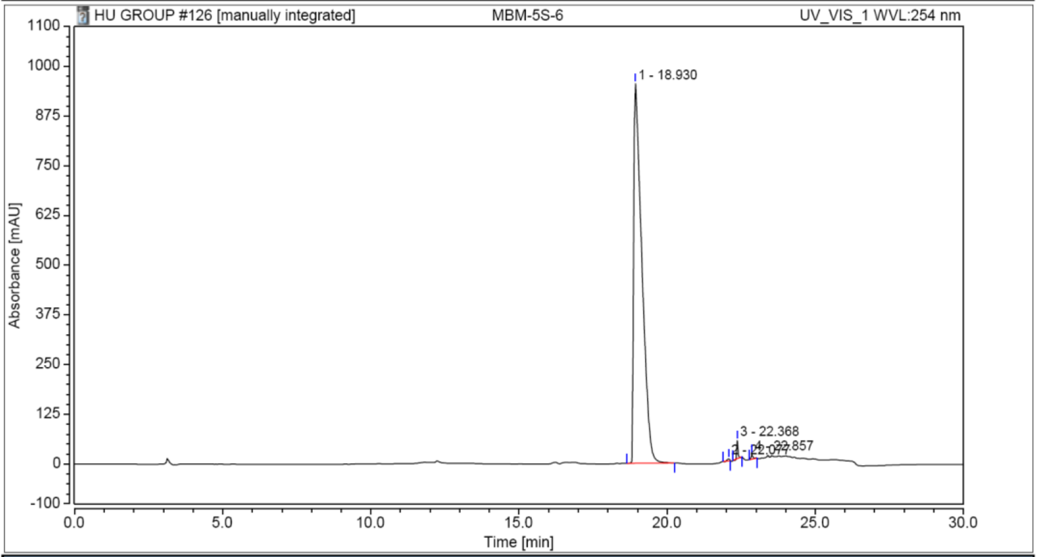

Integration Results

No.	Peak Name	Retention Time<br>min	Area<br>mAU*min	Height<br>mAU	Relative Area<br>%	Relative Height<br>%	Amount<br>n.a.
1		18.930	303.538	954.587	99.02	93.81	n.a.
2		22.077	0.452	5.443	0.15	0.53	n.a.
3		22.368	1.694	45.673	0.55	4.49	n.a.
4		22.857	0.869	11.820	0.28	1.16	n.a.
**Total:**			**306.553**	**1017.524**	**100.00**	**100.00**	

The succinic acid salt of MBM-5: ^{1}H NMR (400 MHz, MeOD) δ 8.39 (dd, J = 7.3, 0.6 Hz, 1H), 8.28 (s, 1H), 8.13 (d, J = 8.1 Hz, 1H), 8.06 (s 1H), 7.81 (m, 2H), 7.77-7.75 (m, 1H), 7.73 (s, 1H), 7.69 (d, J = 7.7 Hz, 1H), 7.59 (t, J = 7.6 Hz, 1H), 7.39 (dd, J = 8.1, 1.5 Hz, 1H), 7.32 (d, J = 1.3 Hz, 1H), 7.21 (dd, J = 7.3, 1.7 Hz, 1H), 5.57 (s, 2H), 4.53 (t, J = 6.2 Hz, 2H), 3.36 (t, J = 6.2 Hz, 2H), 2.70 (s, 6H), 2.54 (s, 8H).

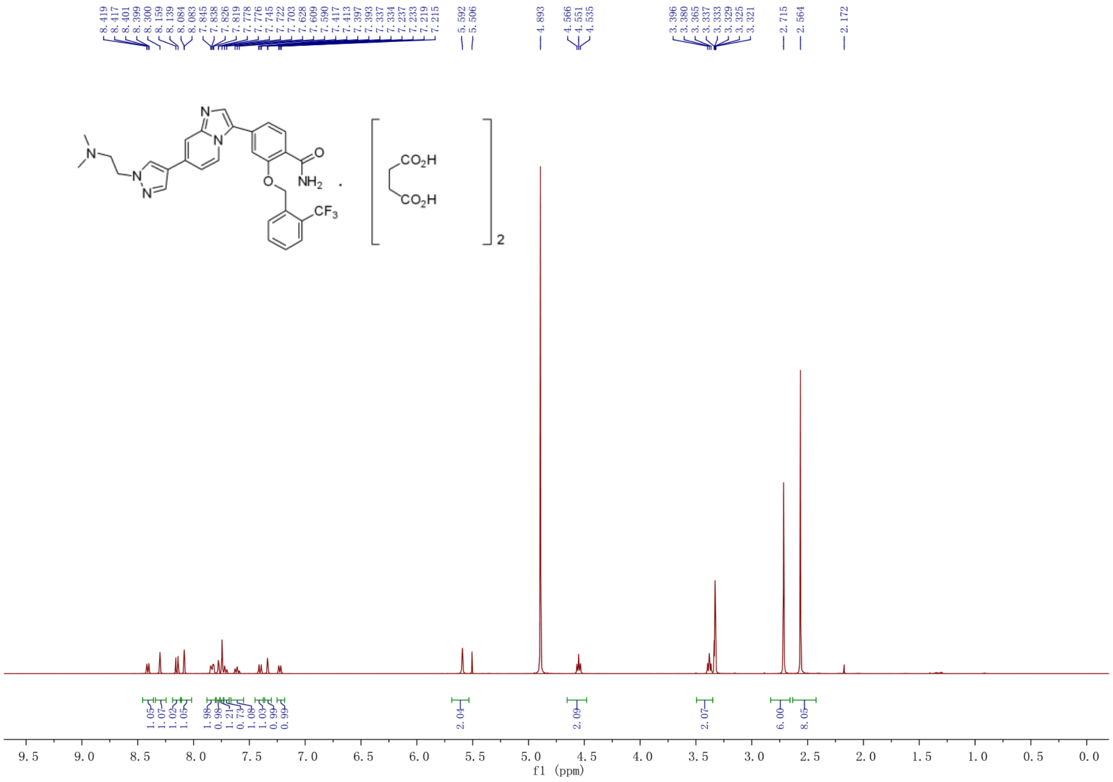

The succinic acid salt of MBM-5: ^{13}C NMR (100 MHz, MeOD) δ 177.40, 169.33, 158.36, 148.23, 138.86, 135.64, 134.83, 133.99, 133.68, 133.60, 132.24, 131.39, 130.13, 129.11 (q, J = 30.8 Hz), 127.50 (q, J = 5.7 Hz), 126.19, 125.85 (q, J = 271.5 Hz), 125.54, 122.83, 122.27, 121.16, 113.55, 113.08, 111.87, 68.84, 58.40, 44.34, 30.95.

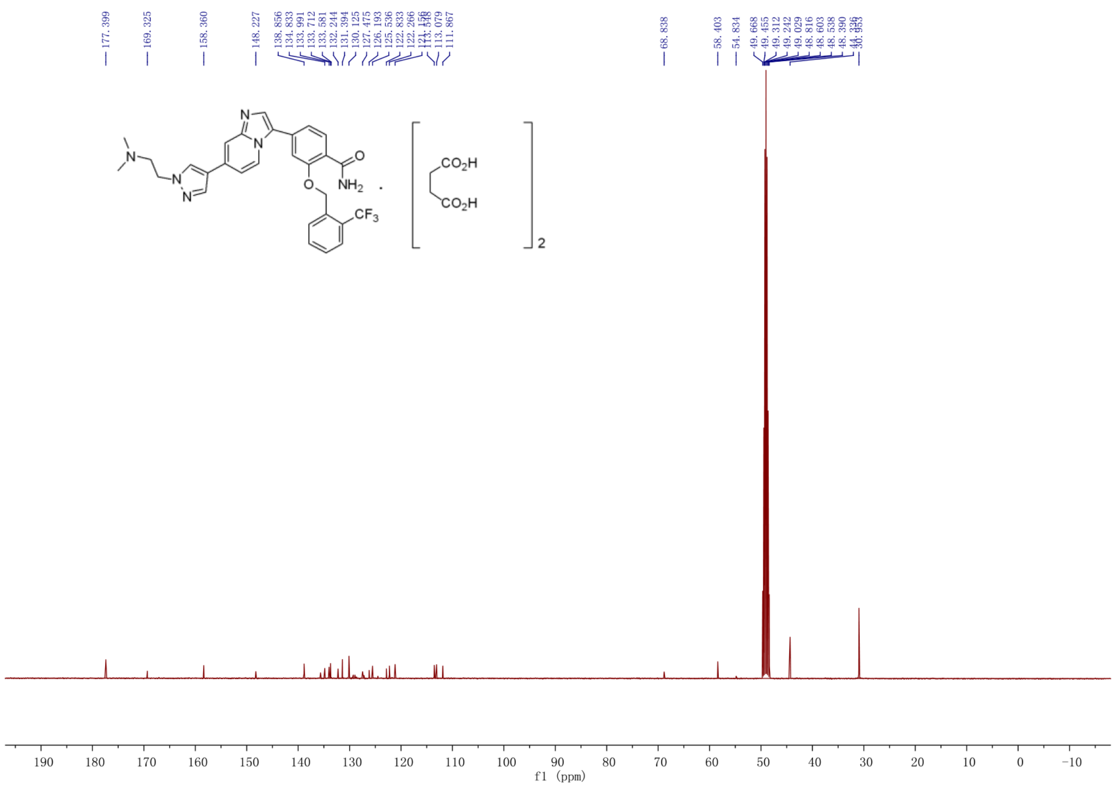

Supplementary Table 1: Pharmacokinetic Parameters obtained after single i.v. administration of 2 mg/kg

Parameter (Mean, n=3)	Values
$T_{1/2}$ (h)	1.4±0.4
MRT_{0-t} (h)	1.2±0.2
AUC_{0-t} (ng/h/mL)	273±26
$AUC_{0-\infty}$ (ng/h/mL)	278±26
CL (ml/h/kg)	121±12
Vdss (L/kg)	8.34±0.74
